# Is Global Limb Anatomic Staging System Classification a Useful Tool in Predicting Lower Limb Revascularization Procedures’ Success?

**DOI:** 10.3390/diseases13030063

**Published:** 2025-02-20

**Authors:** Andreea Luciana Rata, Nawaf Al Khazaleh, Sergiu Sirca, Cătălin Alexandru Pîrvu, Alexandru Furdui, Elena Rizea, Sorin Barac

**Affiliations:** 1 Surgical Emergencies Department, “Victor Babes” University of Medicine and Pharmacy Timisoara, 300041 Timișoara, Romania; andreea.rata@umft.ro (A.L.R.); pirvu.catalin@umft.ro (C.A.P.); 2 Department of Vascular Surgery, Vascular and Endovascular Surgery Research Center, “Pius Brînzeu” Clinical County Emergency Hospital, 300723 Timișoara, Romania; nawaf.al-khazaleh@umft.ro (N.A.K.); sergiusirca867@gmail.com (S.S.); alexandru.furdui@umft.ro (A.F.); elena.rizea@umft.ro (E.R.); 3 Doctoral School, “Victor Babes” University of Medicine and Pharmacy Timisoara, 300041 Timișoara, Romania

**Keywords:** GLASS classification, chronic limb-threatening ischemia, predictability, limb salvage

## Abstract

Background. GLASS (Global Limb Anatomic Staging System) classification is a classification proposed in 2019 by The Lower Extremity Guidelines Committee of the Society for Vascular Surgery, which aims to identify the anatomic substrate that defines the severity of a lower extremity arterial injury and predict the success rate of possible revascularization. The aim of the study is to demonstrate the usefulness of this classification and if it is a reliable tool in predicting the success of the revascularization procedures for patients with chronic limb-threatening ischemia (CLTI). Methods. A retrospective study was conducted on patients undergoing revascularization for CLTI. Glass staging was applied to angiographic data, categorizing them into GLASS 1, 2, or 3 based on the complexity of the femoropopliteal and infrapopliteal lesions. We investigated the clinical characteristics and types of endovascular treatment in correlation with GLASS classification. We also evaluated the technical success of revascularization procedures and the specificity and accuracy of the GLASS classification. Results. After the first testing, we found out that GLASS classification has a sensitivity of 63% and a specificity of 77%. After the second testing, the sensitivity was 82%. of 77% also. The follow-up of this sample was made after 1 year, with no patients lost to follow-up and with an amputation-free survival of 81.3%. Conclusions. GLASS 1 and 2 patients had significantly higher rates of success compared to GLASS 3. GLASS serves as a valuable tool in predicting revascularization success and provides a standardized approach to anatomical complexity, but further studies should integrate more data in order to enhance its predictive capability.

## 1. Introduction

Endovascular surgery has grown remarkably in the last decade, with the proof being that now, globally, this intervention is performed as an elective and as the first intention, with classical methods being reserved for patients who do not have a revascularization solution directed to this type of procedure [1]. The need for a classification to define the revascularization strategy is clear both among experienced surgeons and those just starting their career, which is why the Society for Vascular Surgery, through the Lower Limb Guideline Development Committee, has come to the aid of practitioners by developing the GLASS (Global Limb Anatomic Staging System) Guideline. The classification also incorporates the concept of limb-based patency (LBP), assessing whether an effective target artery path (TAP) can be established to sustain an adequate perfusion post-revascularization [2].

Chronic limb-threatening ischemia (CLTI) represents a severe phase of peripheral arterial disease characterized by rest pain and tissue loss with a high risk of major lower limb amputation [3]. Most of the time arterial lesions are staged suprainguinal, infrainguinal, infragenicular, or inframaleolar, and open and endovascular revascularization strategies should be adopted together [4,5,6].

Despite its potential benefits, the clinical utility of GLASS in predicting revascularization success remains under evaluation. While GLASS provides an anatomical roadmap, it does not integrate hemodynamic parameters, wound severity, or infection status, which are critical in determining patients’ outcomes.

Through this paper, we aim to demonstrate both the usefulness of this classification and the results of its clinical use, analyzing the sample of patients established in the Vascular Surgery Clinic of the County Emergency Hospital “Pius Brînzeu” Timisoara, to use it for the stratification of procedures and approaches to surgical planning. We investigated clinical characteristics, arterial lesions, and types of endovascular treatment according to GLASS classification. We also evaluated the technical success of procedures, events related to revascularized limbs, and the specificity and accuracy of GLASS classification.

By comparing GLASS with other existing classification systems, this study seeks to determine whether GLASS alone is sufficient for guiding revascularization strategies or if it should be integrated with clinical and functional parameters for a more comprehensive approach.

## 2. Materials and Methods

In this study, medical information was collected for the established sample of patients from “Pius Brînzeu” County Emergency Hospital Timisoara. Patients that underwent one of the following procedures were selected: H15101 (percutaneous transluminal balloon angioplasty), H15103 (percutaneous balloon angioplasty with single stenting), and H15104 (percutaneous balloon angioplasty with multiple stenting).

The final sample of patients analyzed in the study met all the selection criteria, being 167 patients who presented from 1 January 2022 to 31 December 2022 to the clinic with PAD (peripheral arterial disease) treated by the mentioned procedures and who were followed up for a period of 12 months (1 January 2023–31 December 2023). All patients gave informed consent for the treatment and for the use of their clinical files under proper anonymization. The data were collected from the patients retrospectively, under the GDPR (General Data Protection Regulation) laws. The study received agreement from the “Pius Brinzeu” Emergency Clinical County Hospital Ethics Committee (no. 500/15 November 2024), under the EU GCP (European Union Good Clinical Practices) Directives, International Conference of Harmonization of Technical Requirements for Registration of Pharmaceuticals for Human Use (ICH) and Declaration of Helsinki.

Patients whose history did not reveal sufficient data to be used by the GLASS Calculator in predicting the difficulty of the intervention and limb patency at 12 months, respectively, were excluded from the final selection. The angiograms were analyzed independently by 3 distinct vascular surgeons and 1 interventional radiologist.

The GLASS Calculator is an application developed by the ESVS Guidelines (European Society of Vascular Surgery) that can be used free of charge and downloaded to a portable device (phone, tablet, etc.). This tool is very useful in this respect as it can calculate a score quickly and easily, even having icons and explanations of the lesions present in the patient’s examination. In the GLASS App calculator (SVS interactive practice guideline (iPG) mobile), we can observe, for example, for a patient with an arterial occlusion that does not allow the infra-malleolar vasculature to perfuse the leg, at the femuropopliteal level, an occlusion >20 cm of which at the popliteal level >5 cm or extended in trifurcation or any popliteal lesion (FP4); in the infrapopliteal segment, it has a diffuse stenosis >2/3 of the total vessel length or chronic total occlusion >1/3 of the vessel (may include the origin of the vessel) or any chronic occlusion of the tibioperoneal trunk (IP4). The presented patient has a GLASS III classification grade, which, according to the classification, indicates a >20% chance of technical failure with a limb patency at one year of <50%. In this case, the anatomic description is as follows: extensive femoropopliteal occlusion or infrapopliteal occlusion, alone or in combination with any lesion in other segments, with chronic popliteal occlusion.

Within the classification, there are 3 categories of lesions that can be categorized for endovascular treatment, grades I/II/III (Table 1):

- Grade I: mild complexity—<10% technical failure and >70% chance of limb patency at 12 months
- Grade II: medium complexity—<20% technical failure and 50–70% chance of limb patency at 12 months
- Grade III: high complexity—>20% technical failure and <50% chance of limb patency at 12 months.
- The definitions of these stages are:
- Stages I and II predict a patency of >70%, respectively, with 50–70% thus defining patients who after an endovascular procedure should not return to the clinic for treatment on the same limb for a period of at least one year,
- Stage III predicts a patency of <50%; patients categorized with this stage have a high chance of returning for repeat procedures on the affected limb.

**Figure 1:**
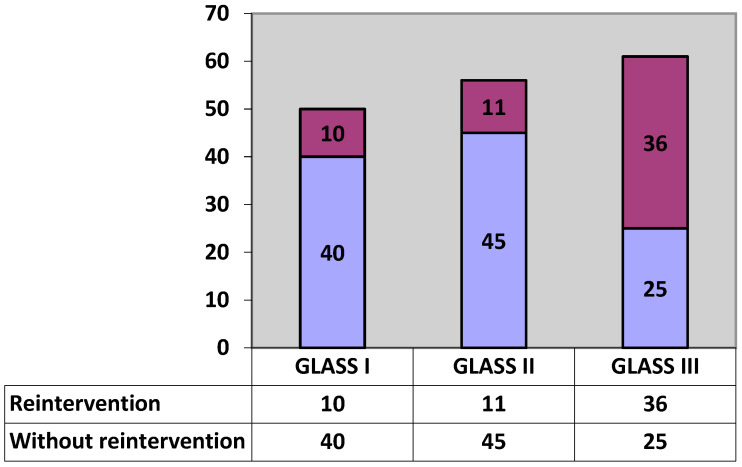
GLASS distribution of the studied sample before first testing.

Patients treated in the study were categorized as follows (Figure 1): 

Patients who were categorized as stage III and for the subsequent 12-month period returned for treatment were considered as affirmative for GLASS predictability of <50% limb patency,

Patients who were stage III and for the subsequent 12-month period did not return for treatment were considered negative for the GLASS prediction of <50% limb patency, and

Patients who were classified as stage I and II and did not return for treatment within the next 12 months were considered positive for the GLASS prediction of >70% and 50–70% limb patency.

Patients who were classified as stage I and II and for the subsequent 12-month period returned for treatment were considered negative for the GLASS prediction of >70% and 50–70% limb patency.

Data were expressed as mean ± standard deviation (SD) for continuous variables with normal distribution and as median [interquartile range] for continuous variables without normal distribution. Categorical variables were expressed as counts and percentages. The overall survival rate and amputation-free survival rate were estimated by the Kaplan–Meier method. A *p*-value < 0.05 was considered statistically significant.

Microsoft Excel and MedCalc version 20.218 were used for all statistical data in the study.

## 3. Results

In total, 167 patients with peripheral artery disease were included in the study, ranging in age from 40 to 92 years old with a mean age of 68 years old (122 men (73.05%) and 45 women (26.95%)).

Analyzing the proportion of procedures performed, it can be seen that 58.1% of the patients treated were treated with procedure H15101 (transluminal balloon angioplasty) for a total of 97 patients (of which 25 were women and 72 were men), respectively, 28.7% with procedure H15103 (percutaneous balloon angioplasty with single stenting) for 48 patients (14 women and 34 men) and 13.2% with procedure H15104 (percutaneous balloon angioplasty with multiple stenting) for 22 patients (6 women and 16 men).

Among the 167 patients, there are 13 patients (7.78%) with I70.21 (atherosclerosis of the arteries of the extremities with intermittent claudication), 35 patients (20.95%) with I70.22 (atherosclerosis of the arteries of the extremities with rest pain), 34 patients (20.35%) with diagnosis I70.23 (atherosclerosis of the extremity arteries with ulceration), and 85 patients (50.89%) with I70. 24 (atherosclerosis of the arteries of the extremities with gangrene) (Table 2).

The most important comorbidities were as follows: 138 patients (82.63%) with arterial hypertension, 103 patients (61.67%) with hyperlipidemia, 95 patients (56.88%) with diabetes mellitus, 84 patients (50.29%) with cardiac insufficiency, and 37 (22.15%) are active smokers.

Out of the 167 patients hospitalized in the clinics for endovascular treatment, the shortest hospitalization period was 2 days and the longest was 32 days. According to the distribution of patients, a median of 5 days to discharge is observed. Regarding the majority distribution of patients, we mention that it is between 2 and 5 days, which reveals the usefulness of the treatment in terms of the hospitalization period as more effective. The shortest procedure was performed in 92 min (approximately 1.5 h) and the longest within 400 min (approximately 6.5 h), with most procedures being performed in a range of 100–200 min (approximately 1.5–3 h) with a median of 200 min (3 h).

The extreme range values attributed to both hospital length and operative time can be attributed to both intraoperative and patient-related complications given the status of comorbidities and the functional hemodynamic status of the patients.

In order to be able to classify the lesions in terms of predictability for the GLASS classification, we defined the criteria for the correlation of results and definition of data for the contingency table that will define the capabilities of this diagnostic method.

First testing can be observed in Table 3. In order to carry out the predictability testing, the following data exposition logic was considered:

- TP (true-positive)—patients who were positively correlated with the GLASS classification were considered to be in the GLASS III category, which specifies that the chances of re-intervention in the patient at one year is high, with the patency being <50%, thus resulting in 36 patients being considered true-positive.
- FP (false-positive)—patients who were not positively correlated with the GLASS classification were considered to be in the GLASS III category, which specifies that the chances of re-intervention in the patient at one year is high, with a patency of <50% resulting in 25 patients considered false-positive.
- FN (false-negative)—patients who were positively correlated with the GLASS classification were considered to be in the GLASS categories I and II, which specify that the chances of re-intervention in the patient at one year is moderately low, with patency >70% and 50–70%, respectively, a criterion that is invalidated when the patient returns within one year, resulting in 21 patients being considered false-negative.
- TNs (true negatives)—patients who were positively correlated with the GLASS classification were considered to be in the GLASS categories I and II, which specify that the chances of re-intervention for the patient at one year is moderately low, with patency >70% and 50–70%, respectively, a criterion that is validated by the fact that the patient does not return for re-intervention within one year, resulting in 85 patients being considered true negatives.

The test based on the diagnostic test by MedCalc presented reveals the following:

The classification has a sensitivity of 63%,
The specificity is 77%,
The positive predictive value is 34%,
The negative predictive value is 59%,
The test accuracy is 72.45%.

Second testing can be observed in Table 4:

- TP (true-positive)—patients who followed the GLASS III and II classifications, respectively, which means that the one-year revascularization patency should be <50% and 50–70%, respectively, considering that GLASS II may have a 50% chance of patency that the patient will present to the clinic for re-intervention, with those with GLASS III patency being even lower than these, who were considered as true positives for the selection criteria, resulting in a total of 47 patients,
- FP (false-positive)—patients who were categorized as GLASS III, a criterion that considers patients with a one-year limb patency of <50%, should have returned to the clinic for re-intervention, but they did not need a new limb intervention, resulting in a false-positive category of 25 patients,
- FNs (false negatives)—patients who were classified GLASS I, which is defined as >70% limb patency at one year, should not have required re-intervention, but within one year they returned to the clinic for a new limb intervention, resulting in false negatives totaling 10 patients,
- TNs (true negatives)—patients who were classified as GLASS I and II, respectively, which means that the revascularization patency at one year should be >70% and 50–70%, respectively, meaning that re-intervention at one year was unlikely, resulting in the fact that they did not return to the clinic for re-intervention, resulting in the true negative category with a total of 85 patients.

The test based on the diagnostic test by MedCalc presented reveals the following:

- The classification has a sensitivity of 82%,
- The specificity is 77%,
- The test accuracy is 72.45%,
- The prevalence was considered 100% because all of the patients examined presented with the condition at the time of consultation, with the purpose of the test being the correct classification of the lesion not its presence.

**Figure 2:**
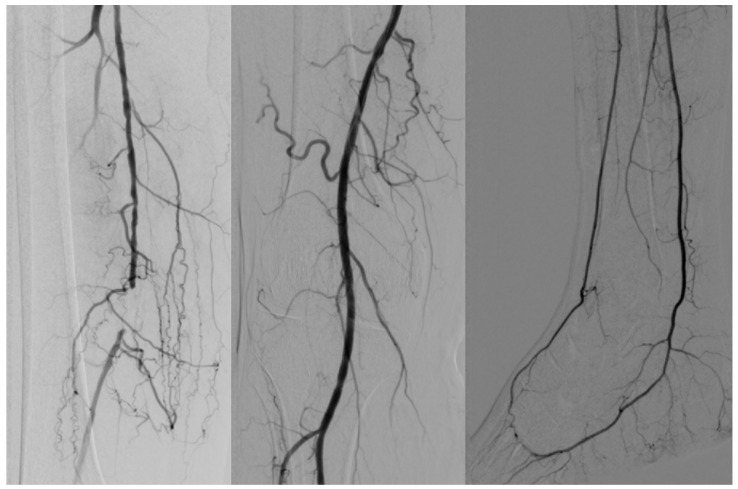
Short occlusion in the distal femoral territory. Ballon angioplasty at this level with complete revascularization of the infrapopliteal segment (GLASS I).

**Figure 3:**
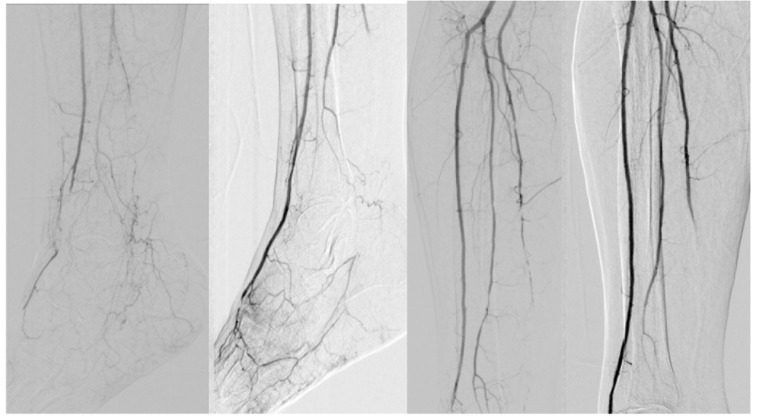
Occlusion of the distal anterior tibial artery (dorsalis pedis artery). Lessions solved through ballon angioplasty (GLASS I).

**Figure 4:**
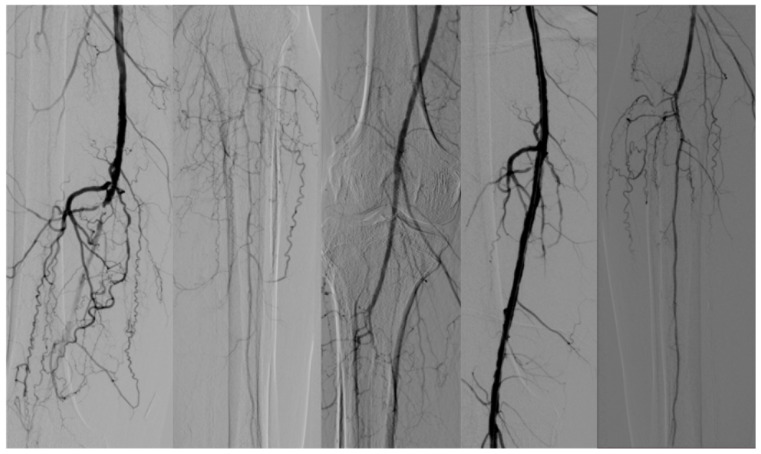
Occlusion of BTK arteries. Revascularization with full recanalization of peroneal artery (GLASS III).

According to the biostatistical criteria of a conventional screening test, the sensitivity value of 82% is considered significant for the presented test, and in terms of accuracy, 72% can be considered a significant result for the presented test. Figure 2, Figure 3 and Figure 4 reveals some of the cases treated.

Another defining factor of this classification is the ability to estimate the complexity of the intervention in terms of the technique used and the anatomical approach that the surgeon will perform based on the imaging data presented on the angiography; as a result, GLASS defines the following:

- Stage I: mild complexity—technical failure <10%,
- Stage II: medium complexity—technical failure <20%,
- Stage III: high complexity—technical failure >20%.

The data presented reveal that:

- Of the 50 patients categorized as having GLASS I, 40 did not require re-intervention, representing a cumulative 80%, compared to 10 patients who required re-intervention, with a cumulative 20%,
- Of the 56 patients categorized as GLASS II, 45 did not require re-intervention, representing a cumulative 80.35%, compared to 11 patients who required re-intervention, with a cumulative 19.65%,
- Of the 61 patients classified as GLASS grade III—36 of these patients had a need for re-intervention, representing a cumulative 59%, compared to 25 patients who did not have a need for re-intervention, with a cumulative 41%.

The follow-up of this sample was made at 3, 6, and 12 months. We have no patients lost to follow-up in our sample. For the entire sample, the Kaplan–Meier overall survival and amputation-free survival for the entire sample were 81.3% and 72.4%, respectively. The GLASS stage 3 was associated with a poor outcome in our sample, and staging in patients in GLASS 3 was an independent predictor of major libm events in these patients.

## 4. Discussion

The study followed a sample of 167 patients from a single academic center; for better evidence of the usefulness of the classification, a study conducted in multiple centers with a common base and a one-year follow-up by a larger group of practitioners with experience in multiple relevant cases would be needed.

The study analyzes aspects of classification for the lower anatomic segment of the femoral artery; it can be extended to the upper segments or upper limbs and follow-up of patency in a segment other than the one analyzed.

The GLASS (Global Limb Anatomic Staging System) classification was proposed in 2019 by The Lower Extremity Guidelines Committee of the Society for Vascular Surgery, which aims to identify the anatomic substrate that defines the severity of a lower extremity injury and predict the success rate of possible revascularization. The definition criteria that are analyzed in this classification are as follows: the hemodynamics of the affected segment (degree of stenosis), and the different anatomical identifications of the femuro-popliteal and infra-popliteal segments, described as two areas of lesions. By this anatomic separation, the surgeon will identify the possible approach of the endovascular procedure following angiography (TAP—target artery pathway) [2,5].

These classification criteria define the degree of complexity of the lesions by diagnosing 3 grades: I—minimal degree of complexity, II—intermediate degree of complexity, and III—major degree of complexity, which can be correlated with the immediate patency of endovascular treatment as well as with the present patency through the viability of the limb 1 year after the intervention [2].

This type of patency corresponds to the healing of the lesions produced by the ischemic obstruction but also the possibility of predicting a future major amputation. With this prognostic tool, it is possible to make the decision to use the pathway with the least lesions as a guide to the site of stenosis, providing the best endovascular approach correlated with the success of the intervention.

A literature review led by Shirasu et al. demonstrated that the GLASS classification describing the complexity of peripheral arterial disease can help predict technical failure, especially in patients treated endovascularly [7].

The retrospective study led by Tokuda that evaluated the success of endovascular techniques on 400 arterial lesions in 257 patients concluded that GLASS predicts de novo success in patients with CLTI [8].

Other studies have concluded that there is a strong association between GLASS classification, amputation rate, limb salvage rate, and wound healing rate in patients with chronic limb-threatening ischemia [9,10].

The present study demonstrated the ability of the GLASS classification to estimate the severity of vascular lesions in the lower limbs. We demonstrated that this classification has a statistically significant sensitivity of 82% and an accuracy of 72% in terms of predictability of classification for revascularization success.

In terms of the complexity of the endovascular procedures performed and the technical difficulty given by the type of anatomic lesions, these were demonstrated to fall into different grades of the GLASS classification, as follows: stage I: mild complexity—<10% technical failure and >70% chance of limb patency at 12 months; stage II: medium complexity—technical failure <20% and 50–70% chance of patency of the member at 12 months, and stage III: high complexity—>20% technical failure and <50% chance of member patency at 12 months.

A systematic review conducted by Bontinis et al., 2023 on the GLASS staging system demonstrated the accuracy of GLASS in predicting immediate technical failure in all stages but failed to predict stages I and II limb-based patency outcomes. The authors also showed the importance of calcium moderator inclusion [11]. Bontinis also stated that GLASS is an efficacious system that matches the treatment of PAD.

GLASS classification is designed to assess anatomic complexity and endovascular treatment in CLTI patients; it introduces the “limb-based patency” concept and evaluates whether a sufficient blood flow path can be restored via the target artery path. However, it has a set of limitations: it does not directly incorporate clinical factors like wound severity or infection, it is primarily anatomy-focused, requiring imaging-based assessment [12,13].

GLASS classification provides a structured and anatomy-based approach for revascularization planning. On the other hand, it lacks the clinical perspective of the WIfi classification, which is better for amputation risk prediction. Also, GLASS and WIfi are often used together, with GLASS guiding revascularization and WIFi predicting amputation risk. A prospective study conducted by Murugavel et al. aimed to demonstrate a correlation between the two types of classification. They found intermediate to good agreement between the two classifications, and the study demonstrated in the end that both classifications predict the limb salvage rate in patients with CLTI [14].

When speaking about Fontaine and Rutherford classifications compared to GLASS, the first two are purely clinical, and regarding hemodynamic parameters, GLASS focuses on anatomical complexity and remains more useful for selecting revascularization strategies [15].

Regarding the amputation-free survival rate and overall survival rate, our results are comparable with the ones found in the literature [16,17].

In a retrospective evaluation of GLASS in patients from the BASIL-1 trial, GLASS 2 and 3 were associated with immediate technical failure [18,19,20]. Also, in our study the amputation-free survival rate tended to be lower in the first 6 months.

Our study has two limitations: a limited number of patients and the short duration of the study. Although the sample size was smaller than other studies, it aligns with similar research and allowed us to conduct a meaningful analysis. We believe that we had a representative dataset that strengthened the reliability of the findings. Also, we believe that the timeframe we chose aligns with similar studies in the field and adequately captured the key trends in the studied matter.

## 5. Conclusions

The usefulness of this classification as well as the multitude of situations in which it can be accessed are undeniably useful in the training of practitioners whose experience will improve over time. They are even comparable to conventional methods of approaching endovascular lesions, which can certainly make a difference when it comes to the daily practice of any clinician, with an increased emphasis on training those who have not had the experience of performing such cases; especially, those whose increased difficulty may require the assistance of a more experienced guide.

The classification was demonstrated to be reliable in terms of technical failure and limb patency.

## Figures and Tables

**Table 1:** GLASS classification and staging (2).

GLASS STAGES						
Femoro-popliteal Staging	4	III	III	III	III	III
	3	II	II	II	III	III
	2	I	II	II	II	III
	1	I	I	II	II	III
	0	-	I	I	II	III
		0	1	2	3	4
	Infrapopliteal Staging					

**Table 2:** Clinical presentation of the studied sample.

Clinical Presentation	No.	%
I70.21	13	7.78
I70.22	35	20.95
I70.23	34	20.35
I70.24	85	50.89

**Table 3:** First testing.

	Re-Intervention	Without Re-Intervention	
GLASS positive	TP = 36	FP = 25	Total GLASS positive = 61
GLASS negative	FN = 21	TN = 85	Total GLASS negative = 106
	Total = 57	Total = 110	Patients total = 167

**Table 4:** Second testing.

	Re-Intervention	Without Re-Intervention	
GLASS positive	TP = 47	FP = 25	Total GLASS positive = 72
GLASS negative	FN = 10	TN = 85	Total GLASS negative = 95
	Total patients = 57	Total patients = 110	Total patients = 167

## Data Availability

The data are available upon request at the first author.
